# Preparation and Characterization of TiO_2_/g-C_3_N_4_/PVDF Composite Membrane with Enhanced Physical Properties

**DOI:** 10.3390/membranes8010014

**Published:** 2018-03-05

**Authors:** Huiya Wang, Ran Gong, Xinliang Qian

**Affiliations:** 1 School of Environmental Engineering, Nanjing Institute of Technology, Nanjing 211167, China; ran_gong@163.com; 2 Jiangsu Huijin Environmental Protection Technology Co., Ltd., Wuxi 214241, China; qianxiinliang@yeah.net

**Keywords:** g-C_3_N_4_, PVDF membrane, TiO_2_, characterization

## Abstract

TiO_2_/g-C_3_N_4_/PVDF composite membranes were prepared by a phase inversion method. A comparison of the performance and morphology was carried out among pure PVDF, g-C_3_N_4_/PVDF, TiO_2_/PVDF and TiO_2_/g-C_3_N_4_/PVDF composite membranes. The results of permeability and instrumental analysis indicated that TiO_2_ and g-C_3_N_4_ organic-inorganic composites obviously changed the performance and structure of the PVDF membranes. The porosity and water content of 0.75TiO_2_/0.25g-C_3_N_4_/PVDF composite membranes were 97.3 and 188.3 L/(m^2^·h), respectively. The porosity and water content of the 0.75TiO_2_/0.25g-C_3_N_4_ membranes were increased by 20.8% and 27.4%, respectively, compared with that of pure PVDF membranes. This suggested that the combination of organic-inorganic composite with PVDF could remarkably improve UTS, membrane porosity and water content.

## 1. Introduction

The research on membranes has aroused great interest due to their wide applications in environmental protection, chemical purification, electrolytes, substrates, coatings, etc. [1,2,3,4,5,6]. Generally, there are two different types of membrane: inorganic and organic [7,8,9,10]. Poly (vinylidene fluoride) (PVDF), a common organic membrane material with excellent chemical resistance and thermal stability, has become a hot research topic in the membrane industry [11,12]. However, PVDF suffers from several disadvantages, such as low surface energy and strong hydrophobic properties, which largely limit the practical application of PVDF [13,14,15,16].

To date, a variety of strategies have been employed to fabricate hydrophilic PVDF membranes, including coating [17], adsorption [18], plasma treatment [19], blending [20] and surface grafting polymerization [21]. Among these, published research results thus far have been focused on PVDF membranes fabricated by nanoparticle coating, due to the unique electronic, magnetic and optical properties of nanoparticles, which would greatly improve the capabilities of polymers [22,23,24,25]. The nanoparticles introduced into PVDF membranes include polymeric chains and metal oxide. From among the polymeric chains, surface-modified macromolecules have been used as additives in the membrane matrix for anti-fouling applications [26,27,28]. This has been an effective strategy for enhancing anti-fouling properties by mitigating membrane fouling. Generally, coating a thin film and grafting polymer chains on the surface of the membrane have been two typical approaches in previous research [29]. Among metal oxide nanoparticles, TiO_2_ has received the most attention, due to its stability and availability [30,31,32,33]. 

Recently, graphitic carbon nitride (g-C_3_N_4_) has become a promising candidate for photocatalysis due to its low cost, visible-light response, simple synthesis and high chemical stability [34,35,36,37]. To date, there have been several reports on the synthesis and application of g-C_3_N_4_/PVDF membranes [38,39,40]. In our previous report, g-C_3_N_4_/PVDF membrane was fabricated through a phase inversion method. It has been found that dispersing g-C_3_N_4_ into PVDF membrane can change the thermal decomposition process of PVDF membranes. 

In this paper, we propose a facial approach for obtaining TiO_2_/g-C_3_N_4_/PVDF composite membranes by a phase inversion method. It is clearly demonstrated that the TiO_2_ and g-C_3_N_4_ can obviously change the performance and structure of PVDF membranes. Moreover, the effects of organic-inorganic composites on the performance and structure of the PVDF membrane are also investigated.

## 2. Experiment

### 2.1. Materials

Poly(vinylidenefluoride) (PVDF), the membrane material, was purchased from the Shanghai 3F New Materials Co., Ltd., Shanghai, China. *N*,*N*′-dimethylformamide (DMF), Silane coupling agent (SCA), polyethylene glycol (PEG 6000), Rutile TiO_2_ nanoparticles, and Melamine were purchased from Sinopharm chemical reagent Co., Ltd., Shanghai, China. All of the used chemicals were of analytical grade, and were used without further purification. 

### 2.2. Preparation of Carbon Nitride (g-C_3_N_4_)

In a typical synthesis, 20 g melamine was transferred to an alumina crucible with a cover and heated to 550 °C in Ar atmosphere for 2 h with a heating rate of 5 °C/min. After undergoing various reactions at high temperature, a light-yellow powder of g-C_3_N_4_ was finally obtained in the alumina crucible.

### 2.3. Preparation of Membrane

In a typical synthesis p, 0.6 g PFG-6000 was introduced in 21 mL DMF and stirred for 30 min at 50 °C. After that, 3 g PVDF and 1.5 mL SCA was added into the previous solution and stirred for another 30 min to form a homogenous suspension. The right amount of TiO_2_ and g-C_3_N_4_ were added and stirred for 4 h. Finally, the final solution was slowly poured on the near end of the glass and a casting knife was placed on one edge of the glass to cast a membrane with a thickness of 2 mm. The obtained samples were denoted as *x*TiO_2_/*y*g-C_3_N_4_/PVDF membrane, where *x* and *y* refer to the mass ratio of TiO_2_ and g-C_3_N_4_. 

### 2.4. Contact Angle Measurement (Sessile-Drop Method)

Water contact angle was measured with a Data Physics optical contact angle measuring instrument with the droplet size controlled using a Gilmont syringe (Chengde Dingsheng testing machine testing equipment Co., Ltd., Chengde, China). Distilled water was used for analysis. The advancing angle was measured when water was added to a droplet spreading over the membrane surface. Droplets were in placed in contact with the membrane at several different locations on each membrane sample to obtain a series of contact angle pairs. All measurements were carried out at room temperature.

### 2.5. Porosity Measurement

In order to evaluate the porosity of the membranes, the membranes were placed in an air-circulating oven at 60 °C for 24 h. Then, they were weighed after wiping off surface water with blotting paper. After that, the wet membranes were placed in an oven at 80 °C for 24 h in order to ensure they were completely dry. The porosity of the membranes (*P*) was calculated by:

$$P(\%)=\frac{W_{0}-W_{1}}{Ah}\times 1000,$$

where *P* is the porosity of membrane, *W*_0_ is the wet sample weight (g), *W*_1_ is the dry sample weight (g), *A* is the square of membrane (cm^2^) and *h* is the thickness of membrane (mm).

### 2.6. Characterization

The mechanical strength of the membranes was tested by Instron 5542 Material Testing Instrument (Changchun Kexin Experimental Instrument Co., Ltd., Changchun, China) at room temperature (25 °C) and 80% relative humidity. Fourier Transform Infrared (FT-IR) spectroscopy was performed on a Nexus 870 spectrometer (BRUKER, TENSOR27, Karlsruhe, Germany). Field Emission Scanning Electron Microscopy (S-4800 Hitachi, Tokyo, Japan) was applied to observe the morphology of the resulting membranes. Each sample was clamped at the both ends with an initial gauge length of 100 mm and width of 20 mm. Thermogravimetric Analysis (Simultaneous TGA-DSC, New Castle, DE, USA) was conducted under nitrogen from 30 to 700 °C at a heating rate of 10 °C·min^−1^. X-ray diffraction patterns were recorded by X-ray Diffractometer (BRUKER-AXS, Karlsruhe, Germany).

## 3. Results and Discussion

**Figure 1:**
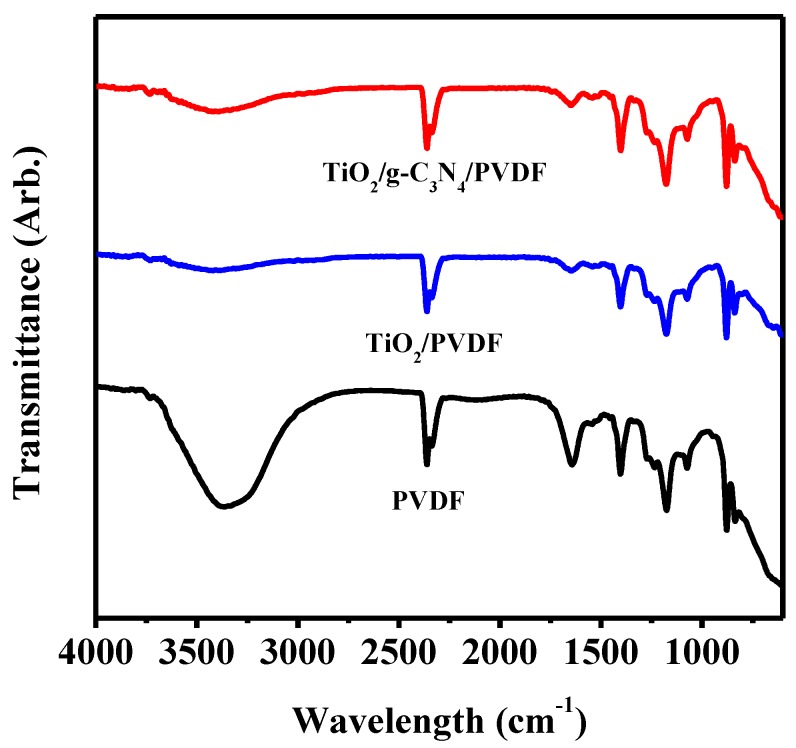
FTIR spectra of PVDF, TiO_2_/PVDF, g-C_3_N_4_/PVDF and TiO_2_/g-C_3_N_4_/PVDF membranes.

Figure 1 depicts the FT-IR spectra of PVDF, TiO_2_/PVDF, g-C_3_N_4_/PVDF and TiO_2_/g-C_3_N_4_/PVDF membranes. The 0.75TiO_2_/0.25g-C_3_N_4_/PVDF membrane was characterized by typical IR patterns of PVDF membrane, indicating that the main chemical skeleton of PVDF membrane had been retained. There is a weak band located at 2917 cm^−1^, which is associated with the CH stretching of PVDF structure [41]. The band at 3341 cm^−1^ is associated with OH stretching vibration of water molecules emanating from the polymer pores [42]. Notably, the absorption band of OH for TiO_2_/PVDF was weakened compared with that of PVDF, which may be caused by the effects of the hydrogen bonds between the fluorine atoms in PVDF and the oxygen atoms in TiO_2_, implying that the TiO_2_ had been successfully distributed on the surface of PVDF. Interestingly, the band at 3341 cm^−1^ appeared in g-C_3_N_4_/TiO_2_/PVDF, which was associated with the stretching mode of N–H of g-C_3_N_4_ [43]. This phenomenon was attributed to the g-C_3_N_4_ having also been successfully distributed on the surface of PVDF.

**Figure 2:**
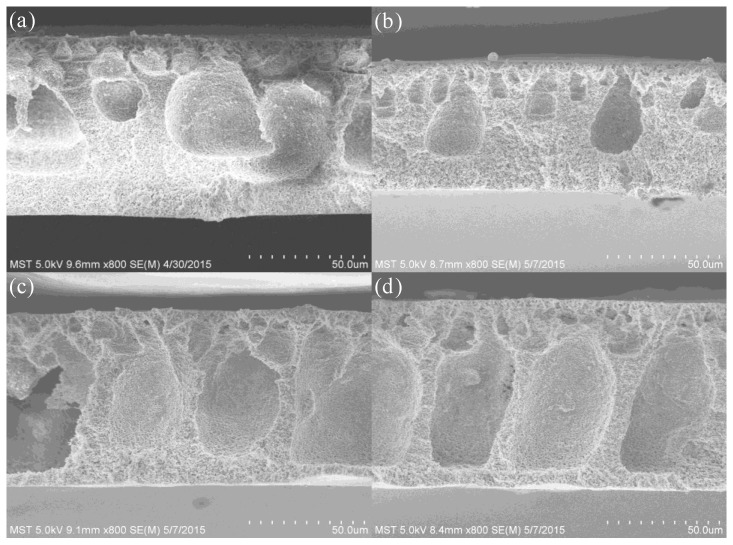
SEM images of (**a**) PVDF membrane; (**b**) TiO_2_/PVDF membrane; (**c**) g-C_3_N_4_/PVDF membrane; and (**d**) TiO_2_/g-C_3_N_4_/PVDF.

The morphologies of PVDF, TiO_2_/PVDF, g-C_3_N_4_/PVDF and g-C_3_N_4_/TiO_2_/PVDF membranes were investigated through SEM, and the results are presented in Figure 2. Before SEM, the sample membranes have to be dried. Generally, three different drying methods have been proposed to dry membranes, including room temperature-oven drying, ethanol-hexane drying, and freeze-drying [44,45]. In this paper, room temperature-oven drying is used. The membranes are firstly dried at room temperature for 12 h, and then dried in an oven of 120 °C for 6 h. Compared with the porous and coarse structure of PVDF membrane (Figure 2a), both TiO_2_/PVDF and g-C_3_N_4_/PVDF (Figure 2b,c) membranes show a smooth structure with circular and dark voids of uniform dimensions. Notably, the g-C_3_N_4_/TiO_2_/PVDF membrane (Figure 2d) shows decreased but more regular and uniform voids. 

To further study the physical strength and durability of the membrane, the Ultimate Tensile Strength (UTS) was measured, and the results are shown in Table 1, Table 2 and Table 3. As expected, 0.75TiO_2_/0.25g-C_3_N_4_/PVDF membrane showed the highest UTS value. Values for TiO_2_/PVDF, g-C_3_N_4_/PVDF, and 0.75TiO_2_/0.25g-C_3_N_4_/PVDF membranes were measured as 7.5, 7.1 and 8.7 MPa, respectively. It is clear that the addition of TiO_2_ and g-C_3_N_4_ to PVDF improved the mechanical strength of the composite membranes. It is well known that membranes with macro-void morphologies often show inferior mechanical properties [42,46]. It was assumed that the increased UTS in TiO_2_/g-C_3_N_4_/PVDF composite membrane was attributable to the decreased voids in the membrane structure. Furthermore, 0.75TiO_2_/0.25g-C_3_N_4_/PVDF composite membrane exhibited the highest tensile strength, which could be attributable to there being fewer voids in the membrane structure.

To determine the hydrophilicity/hydrophobicity of the membrane surface, the contact angle was measured. As shown in Table 1, the contact angle of the as-prepared membrane with TiO_2_ and g-C_3_N_4_ was decreased. It is well known that the membrane wettability of the membrane is influenced by the membrane material, as well as the surface porosity and roughness [47,48]. After adding TiO_2_ and g-C_3_N_4_, the surface porosity sharply increased. In other words, adding the TiO_2_ and g-C_3_N_4_ leads to a higher surface porosity and results in a decreased contact angle of the membrane. 

Membrane porosity and water content play an important role in membrane performance. As shown in Table 1, the porosity and water content of the 0.75TiO_2_/0.25g-C_3_N_4_/PVDF composite membranes were 97.3 and 188.3 L/(m^2^·h), respectively. This can be ascribed to the improvement in the hydrophilicity of the composite membranes because of the addition of TiO_2_ and g-C_3_N_4_.

**Figure 3:**
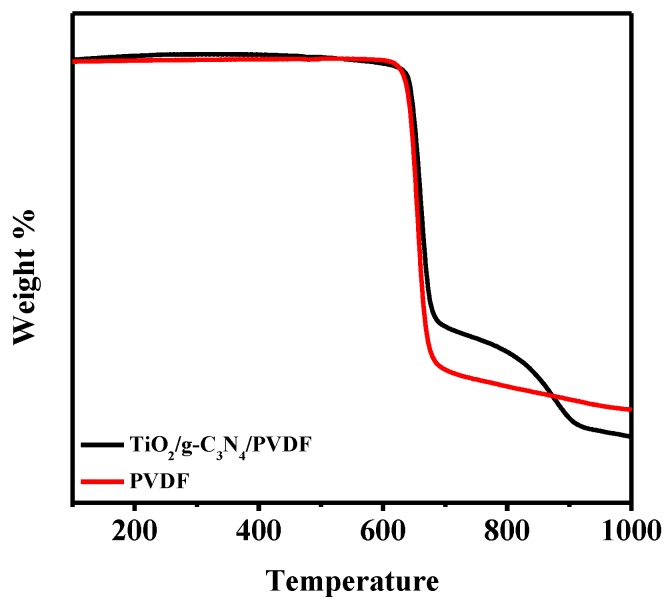
TGA weight loss profiles of PVDF and TiO_2_/g-C_3_N_4_ PVDF.

To further explore the pyrolysis properties of the 0.75TiO_2_/0.25g-C_3_N_4_/PVDF composite membrane, a temperature-domain TGA was conducted, and the results are shown in Figure 3. It is clearly shown that the TiO_2_/g-C_3_N_4_/PVDF composite membrane exhibited a higher thermal decomposition temperature than that of the pure PVDF membrane, indicating the better thermal stability of the composite membrane, which could potentially be attributed to the physical and chemical interactions between PVDF chains and g-C_3_N_4_ surface functional groups.

**Figure 4:**
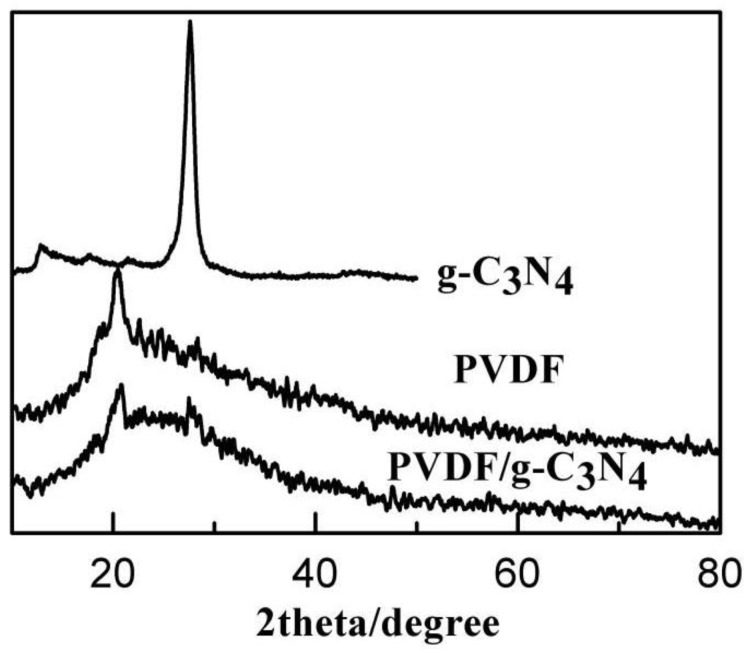
XRD patterns of PVDF, g-C_3_N_4_, and g-C_3_N_4_/PVDF.

The crystal structures of the g-C_3_N_4_, PVDF and g-C_3_N_4_ PVDF membranes were characterized by XRD. As shown in Figure 4, two pronounced peaks at around 13.0° and 27.4° were observed in the XRD patterns of g-C_3_N_4_, corresponding to the in-plane structural packing motif and the interlayer stacking of aromatic systems, respectively [34]. The XRD patterns of the g-C_3_N_4_/PVDF membrane showed a weak peak of g-C_3_N_4_, which could be attributed to the low g-C_3_N_4_ content. 

## 4. Conclusions

A TiO_2_/g-C_3_N_4_/PVDF composite membrane was fabricated by a phase inversion method. A comparison of the performance and morphology was carried out between pure PVDF membrane and PVDF composite membranes with different mass ratios of TiO_2_ and g-C_3_N_4_. It was clearly shown that the TiO_2_ and g-C_3_N_4_ were able to obviously change the performance and structure of PVDF membranes. Contact angle measurements and SEM images showed that the addition of 0.75 g TiO_2_ and 0.25 g g-C_3_N_4_ to PVDF membrane greatly improved hydrophilicity due to the decreased number of voids. The porosity and water content of the 0.75TiO_2_/0.25g-C_3_N_4_/PVDF composite membranes were 97.3 and 188.3 L/(m^2^·h), respectively. The porosity and water content of the 0.75TiO_2_/0.25g-C_3_N_4_ membrane were increased by 20.8% and 27.4%, respectively, compared with those of the pure PVDF membrane. These results provide a novel way of improving membrane morphology, structure and stability without influencing the separation function.

## Figures and Tables

**Table 1:** TiO_2_/g-C_3_N_4_/PVDF composite membranes compositions.

Membrane	Composition (wt %)	UTS (MPa)	Contact Angle	Porosity	Water Content
PVDF	-	0.27	75.98	50	90.3
TiO_2_/PVDF	-	0.29	56.22	71	111.3
g-C_3_N_4_/PVDF	-	0.29	58.64	71	127.7
TiO_2_/g-C_3_N_4_/PVDF	0.75:0.25	0.33	62.57	67	143.2
TiO_2_/g-C_3_N_4_/PVDF	0.5:0.5	0.29	70.89	60	124.5

**Table 2:** Water content of TiO_2_/g-C_3_N_4_/PVDF composite membrane.

Parametric Membrane	Composition (wt %)	Contact Angle			Water Content		
		Initial State	30 s	60 s	Container Quality	3 min Later Quality	*J*_W_ L/(m^2^·h)
PVDF	-	82.34	72.62	75.98	1.9164	7.7294	90.3
TiO_2_/PVDF	-	73.27	68.06	56.22	4.0196	10.6646	111.3
g-C_3_N_4_/PVDF	-	75.53	65.94	58.64	3.9521	11.5824	127.7
TiO_2_/g-C_3_N_4_/PVDF	0.75:0.25	79.29	70.93	62.57	3.8871	12.4376	143.2
TiO_2_/g-C_3_N_4_/PVDF	0.5:0.5	77.53	79.42	70.89	2.7236	9.8236	124.5

**Table 3:** Porosity of TiO_2_/g-C_3_N_4_/PVDF composite membrane.

Parametric Membrane	Composition (wt %)	Porosity (%)		
		*m*_wet_ (g)	*m*_dry_ (g)	*ε*
PVDF	-	0.06	0.03	50%
TiO_2_/PVDF	-	0.07	0.02	71%
g-C_3_N_4_/PVDF	-	0.07	0.02	71%
TiO_2_/g-C_3_N_4_/PVDF	0.75:0.25	0.06	0.02	67%
TiO_2_/g-C_3_N_4_/PVDF	0.5:0.5	0.05	0.02	60%

## References

[1] Shu C., Song B., Wei X., Liu Y., Tan Q., Chong S., Chen Y., Yang X.-D., Yang W.-H., Liu Y. (2018). Mesoporous 3D nitrogen-doped yolk-shelled carbon spheres for direct methanol fuel cells with polymer fiber membranes. Carbon.

[2] Colò F., Bella F., Nair J.R., Gerbaldi C. (2017). Light-cured polymer electrolytes for safe, low-cost and sustainable sodium-ion batteries. J. Power Sources.

[3] Bella F., Pugliese D., Zolin L., Gerbaldi C. (2017). Paper-based quasi-solid dye-sensitized solar cells. Electrochim. Acta.

[4] Hu E.N., Lin C.X., Liu F.H., Wang X.Q., Zhang Q.G., Zhu A.M., Liu Q.L. (2018). Poly(arylene ether nitrile) anion exchange membranes with dense flexible ionic side chain for fuel cells. J. Membr. Sci..

[5] Nair J.R., Bella F., Angulakshmi N., Stephan A.M., Gerbaldi C. (2016). Nanocellulose-laden composite polymer electrolytes for high performing lithium–sulphur batteries. Energy Storage Mater..

[6] Bella F., Ozzello E.D., Bianco S., Bongiovanni R. (2013). Photo-polymerization of acrylic/methacrylic gel–polymer electrolyte membranes for dye-sensitized solar cells. Chem. Eng. J..

[7] Jha R., Dulikravich G.S., Chakraborti N., Fan M., Schwartz J., Koch C.C., Colaco M.J., Poloni C., Egorov I.N. (2016). Algorithms for design optimization of chemistry of hard magnetic alloys using experimental data. J. Alloys Compd..

[8] Fan M., Liu Y., Jha R., Dulikravich G.S., Schwartz J., Koch C.C. (2016). On the Formation and Evolution of Cu-Ni-Rich Bridges of Alnico Alloys with Thermomagnetic Treatment. IEEE Trans. Magn..

[9] Wang R., Yang C., Fan M., Wu M., Wang C., Yu X., Zhu J., Zhang J., Li G., Huang Q. (2013). Phase relationship of the TbO_1.81_–Mn_3_O_4_–Fe_2_O_3_ system synthesized at 1200 °C. J. Alloys Compd..

[10] Fan M., Liu Y., Jha R., Dulikravich G.S., Schwartz J., Koch C.C. (2016). On the evolution of Cu-Ni-rich bridges of Alnico alloys with tempering. J. Magn. Magn. Mater..

[11] Yeow M.L., Liu Y., Li K. (2005). Preparation of porous PVDF hollow fibre membrane via a phase inversion method using lithium perchlorate (LiClO4) as an additive. J. Membr. Sci..

[12] Yu L.-Y., Xu Z.-L., Shen H.-M., Yang H. (2009). Preparation and characterization of PVDF–SiO_2_ composite hollow fiber UF membrane by sol–gel method. J. Membr. Sci..

[13] Cha S., Kim S.M., Kim H., Ku J., Sohn J.I., Park Y.J., Song B.G., Jung M.H., Lee E.K., Choi B.L. (2011). Porous PVDF as Effective Sonic Wave Driven Nanogenerators. Nano Lett..

[14] Wu C.M., Chou M.H. (2016). Polymorphism, piezoelectricity and sound absorption of electrospun PVDF membranes with and without carbon nanotubes. Compos. Sci. Technol..

[15] Zaddach A.J., Niu C., Oni A.A., Fan M., LeBeau J.M., Irving D.L., Koch C.C. (2016). Structure and magnetic properties of a multi-principal element Ni–Fe–Cr–Co–Zn–Mn alloy. Intermetallics.

[16] Zhang G., Fan M. (2011). ChemInform Abstract: Synthesis and Magnetic Properties of Double B Mixed Perovskite Series La_0.75_K_0.25_Mn_1−x_FexO_3_. ChemInform.

[17] Sahoo B.N., Balasubramanian K. (2014). Facile synthesis of nano cauliflower and nano broccoli like hierarchical superhydrophobic composite coating using PVDF/carbon soot particles via gelation technique. J. Colloid Interface Sci..

[18] Dang Z.-M., Wang H.-Y., Zhang Y.-H., Qi J.-Q. (2005). Morphology and Dielectric Property of Homogenous BaTiO_3_/PVDF Nanocomposites Prepared via the Natural Adsorption Action of Nanosized BaTiO_3_. Macromol. Rapid Commun..

[19] Li M., Shi J., Chen C., Li N., Xu Z., Li J., Lv H., Qian X., Jiao X. (2017). Optimized permeation and antifouling of PVDF hybrid ultrafiltration membranes: Synergistic effect of dispersion and migration for fluorinated graphene oxide. J. Nanopart. Res..

[20] Liu J., Tian C., Xiong J., Wang L. (2017). Polypyrrole blending modification for PVDF conductive membrane preparing and fouling mitigation. J. Colloid Interface Sci..

[21] Shen L., Feng S., Li J., Chen J., Li F., Lin H., Yu G. (2017). Surface modification of polyvinylidene fluoride (PVDF) membrane via radiation grafting: Novel mechanisms underlying the interesting enhanced membrane performance. Sci. Rep. UK.

[22] Khayet M., Villaluenga J., Valentin J.L., Lopez-Manchado M., Mengual J.I., Seoane B. (2005). Filled Poly(2,6-dimethyl-1,4-phenylene Oxide) Dense Membranes by Silica and Silane Modified Silica Nanoparticles: Characterization and Application in Pervaporation. Polymer.

[23] Liu Z., Pang L., Li Q., Zhang S., Li J., Tong H., Xu Z., Yi C. (2017). Hydrophilic porous polyimide/β-cyclodextrin composite membranes with enhanced gas separation performance and low dielectric constant. High Perform. Polym..

[24] Jia H., Wu Z., Liu N. (2017). Effect of nano-ZnO with different particle size on the performance of PVDF composite membrane. Plast. Rubber Compos..

[25] Albo J., Santos E., Neves L.A., Simeonov S.P., Afonso C.A.M., Crespo J.G., Irabien A. (2012). Separation performance of CO_2_ through Supported Magnetic Ionic Liquid Membranes (SMILMs). Sep. Purif. Technol..

[26] Rana D., Matsuura T. (2010). Surface Modifications for Antifouling Membranes. Chem. Rev..

[27] Manawi Y., Kochkodan V., Mahmoudi E., Johnson D.J., Mohammad A.W., Atieh M.A. (2017). Characterization and Separation Performance of a Novel Polyethersulfone Membrane Blended with Acacia Gum. Sci. Rep. UK.

[28] Rana D., Kim Y., Matsuura T., Arafat H.A. (2011). Development of antifouling thin-film-composite membranes for seawater desalination. J. Membr. Sci..

[29] Shahkaramipour N., Tran T., Ramanan S., Lin H. (2017). Membranes with Surface-Enhanced Antifouling Properties for Water Purification. Membranes.

[30] Cao X., Ma J., Shi X., Ren Z. (2006). Effect of TiO_2_ nanoparticle size on the performance of PVDF membrane. Appl. Surf. Sci..

[31] Wang X., Xinxin Zhao C., Xu G., Chen Z.-K., Zhu F. (2012). Degradation mechanisms in organic solar cells: Localized moisture encroachment and cathode reaction. Sol. Energy Mater. Sol. C.

[32] Chen J., Zhao C.X., Zhi M.M., Wang K., Deng L., Xu G. (2012). Alkaline direct oxidation glucose fuel cell system using silver/nickel foams as electrodes. Electrochim. Acta.

[33] Deng L.L., Zhao C.X., Ma Y., Chen S.S., Xu G. (2013). Low cost acetone sensors with selectivity over water vapor based on screen printed TiO2 nanoparticles. Anal. Methods.

[34] Cao S., Zhou N., Gao F., Chen H., Jiang F. (2017). All-solid-state Z-scheme 3,4-dihydroxybenzaldehyde-functionalized Ga_2_O_3_/graphitic carbon nitride photocatalyst with aromatic rings as electron mediators for visible-light photocatalytic nitrogen fixation. Appl. Catal. B Environ..

[35] Samanta S., Khilari S., Pradhan D., Srivastava R. (2017). An Efficient, Visible Light Driven, Selective Oxidation of Aromatic Alcohols and Amines with O_2_ Using BiVO_4_/g-C_3_N_4_ Nanocomposite: A Systematic and Comprehensive Study toward the Development of a Photocatalytic Process. ACS Sustain. Chem. Eng..

[36] Giannakopoulou T., Papailias I., Todorova N., Boukos N., Liu Y., Yu J., Trapalis C. (2017). Tailoring the energy band gap and edges’ potentials of g-C_3_N_4_/TiO_2_ composite photocatalysts for NOx removal. Chem. Eng. J..

[37] Zhao C.X., Wang X., Zeng W., Chen Z.K., Ong B.S., Wang K., Deng L., Xu G. (2011). Organic photovoltaic power conversion efficiency improved by AC electric field alignment during fabrication. Appl. Phys. Lett..

[38] Wang H.Y., Hang Z.S., Lu X.M., Ying S.J. (2013). Preparation and pyrolysis performance of g-C_3_N_4_/PVDF composite membrane. Mod. Chem. Ind..

[39] Yan H., Zhao C., Wang K., Deng L., Ma M., Xu G. (2013). Negative dielectric constant manifested by static electricity. Appl. Phys. Lett..

[40] Zhao C.X., Mao A.Y., Xu G. (2014). Junction capacitance and donor-acceptor interface of organic photovoltaics. Appl. Phys. Lett..

[41] Thomas E., Parvathy C., Balachandran N., Bhuvaneswari S., Vijayalakshmi K.P., George B.K. (2017). PVDF-ionic liquid modified clay nanocomposites: Phase changes and shish-kebab structure. Polymer.

[42] Adams F.V., Nxumalo E.N., Krause R.W.M., Hoek E.M.V., Mamba B.B. (2012). Preparation and characterization of polysulfone/β-cyclodextrin polyurethane composite nanofiltration membranes. J. Membr. Sci..

[43] Yan J., Rodrigues M.-T.F., Song Z., Li H., Xu H., Liu H., Wu J., Xu Y., Song Y., Liu Y. (2017). Reversible Formation of g-C_3_N_4_ 3D Hydrogels through Ionic Liquid Activation: Gelation Behavior and Room-Temperature Gas-Sensing Properties. Adv. Funct. Mater..

[44] Albo J., Wang J., Tsuru T. (2014). Gas transport properties of interfacially polymerized polyamide composite membranes under different pre-treatments and temperatures. J. Membr. Sci..

[45] Albo J., Hagiwara H., Yanagishita H., Ito K., Tsuru T. (2014). Structural Characterization of Thin-Film Polyamide Reverse Osmosis Membranes. Ind. Eng. Chem. Res..

[46] Ogawa H., Kanaya T., Nishida K., Matsuba G. (2008). Phase separation and dewetting in polystyrene/poly(vinyl methyl ether) blend thin films in a wide thickness range. Polymer.

[47] Jiang J.-H., Zhu L.-P., Li X.-L., Xu Y.-Y., Zhu B.-K. (2010). Surface modification of PE porous membranes based on the strong adhesion of polydopamine and covalent immobilization of heparin. J. Membr. Sci..

[48] McCutcheon J.R., Elimelech M. (2008). Influence of membrane support layer hydrophobicity on water flux in osmotically driven membrane processes. J. Membr. Sci..

